# The analgesic and antidepressant properties of dihydroartemisinine in the neuropathic pain mice: By the downregulation of HnRNPA1 in the spinal cord and hippocampus

**DOI:** 10.1002/ctm2.751

**Published:** 2022-02-27

**Authors:** Chunyan Zhu, Yongping Zhu, Guoxin Zhang, Hongyan Wu, Yuqi Shi, Jiahao Li, Jun Yang, Zhiyun Mao, Qionghong Xu, Xuemin Yao, Xiaoxin Zhu, Jigang Wang, Xianguo Liu, Na Lin

**Affiliations:** ^1^ Institute of Chinese Materia Medica China Academy of Chinese Medical Sciences Beijing China; ^2^ Artemisinin Research Center and Institute of Chinese Materia Medica China Academy of Chinese Medical Sciences Beijing China; ^3^ Department of Physiology and Pain Research Center Zhongshan School of Medicine Sun Yat‐sen University Guangzhou 510080 China

**FIGURE 1:**
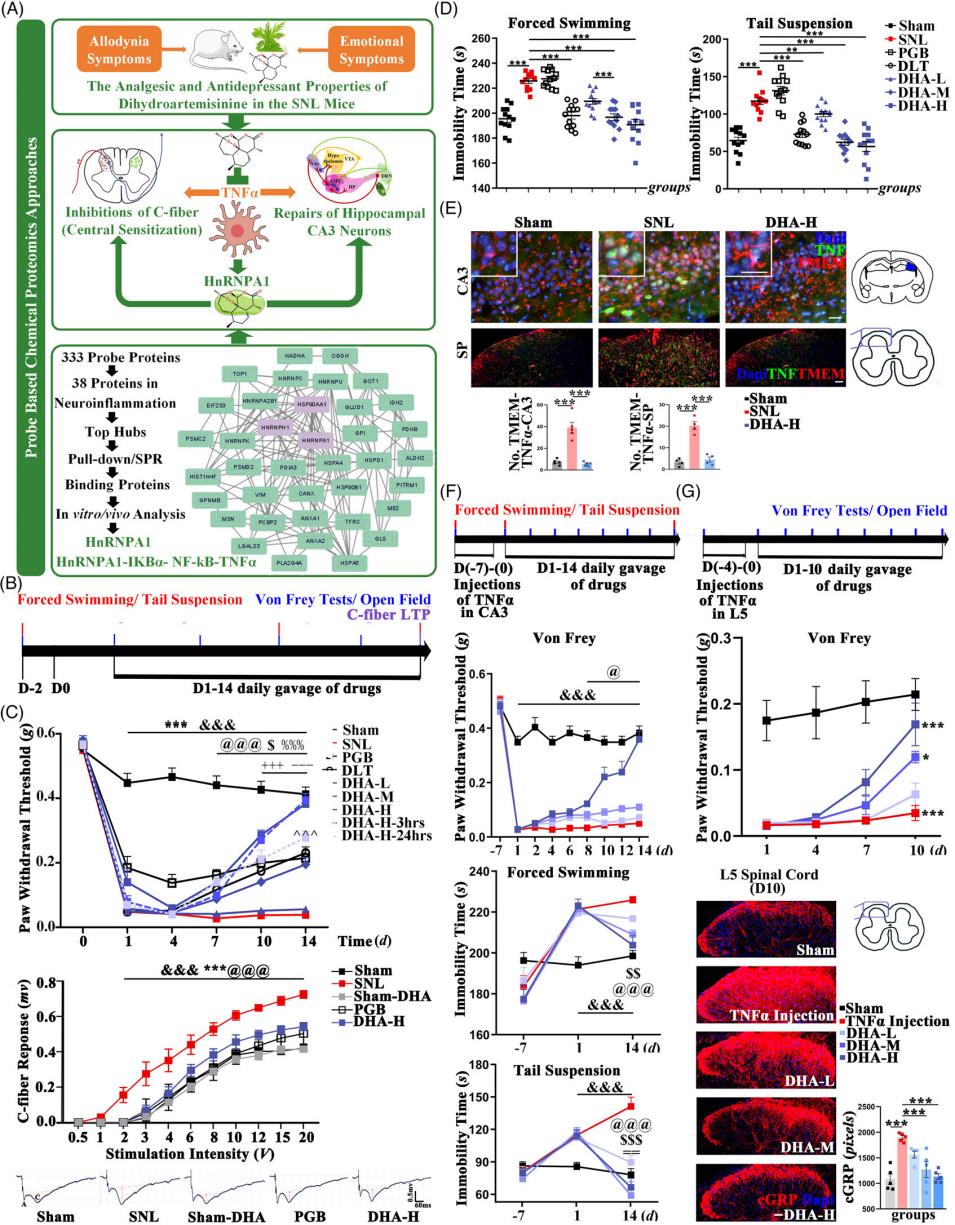
The graphical abstract and the clarification of the pain‐emotion co‐curations by DHA in both the spinal cord ligation (SNL) and TNFα injected mice. (A) The graph describing the contents of this study. (B) The time axis of the tests in the SNL mice. The grouped data (mean ±S.E.M, *n* = 12 mice per group) show (C) the allodynia behaviors and the C‐fiber long‐term potentiation (LTP), (D) the depression behaviours, (E) and the expression of TNFα in the CA3 (scale bar 50 μm, *n* = 5 mice per group) and SP (scale bar 50 μm, *n* = 4 mice per group); consequences of the consecutive injections of TNFα in the CA3 and SP were quantified as follows. (F) In CA3 injected ones, the allodynia, depression behaviours (*n* = 8 mice per group) were assessed. (G) In SP injected ones, the allodynia behaviours (*n* = 8 mice per group), the expression of cGRP in the dorsal horn of the L5 spinal cord (scale bar 50 μm, *n* = 5 mice per group) were assessed. In B–C–F, ^&&&^denotes the significant difference between Sham and SNL/TNFα injection (*P* < 0.001), ***denotes significant difference between SNL and PGB (*P* < 0.001), ^%%%, %%^denotes significant difference between SNL and DLT (*P* < 0.001, *P* < 0.01), ^@@@,@@,@^denotes the significant difference between SNL/TNFα injection and DHA‐H (*P* < 0.001, *P* < 0.01, *P* < 0.05), ^$$$,$$,$^denotes the significant difference between SNL/TNFα injection and DHA‐M (*P* < 0.001, *P* < 0.01, *P* < 0.05),^+++^denotes significant difference between DHA‐H and DHA‐M (*P* < 0.001),^==^denotes significant difference between TNFα injection and DHA‐L (P <0.01),^–−^denotes significant difference between DHA‐M and DHA‐L (*P* < 0.001),^∧∧∧^denotes significant difference between DHA‐H and DHA‐H‐24 h (*P* < 0.001). In D–E–G, ***P < 0.001

Dear Editor,

Neuropathic pain (NP) is far from effectively treated. The failures are more associated with the emotional, rather than the sensory aspects.^1^ Therefore, the pain‐emotion co‐curation is of primary importance.^2^ Our team revealed the analgesic and antidepressant properties of dihydroartemisinine (DHA) in the spinal cord ligation (SNL) mice;^3^ clarified that the synergistic regulations of TNFα in the spinal cord (SP) and hippocampus (CA3) may explain for the inhibitions of the C‐fiber long‐term potentiation (LTP) in the SP and the repairs of CA3 pyramidal neurons; identified heterogeneous nuclear ribonucleoprotein A1 (HnRNPA1) as the binding target of DHA, by which DHA regulate the expression of TNFα (Figure 1A).

The analgesic and antidepressant effects were evaluated in the first‐line drug pregabalin (PGB,45 mg/kg), duloxetine (DLT, 9 mg/kg) and DHA (H‐14.5 mg/kg, M‐7.25 mg/kg, L‐3.625 mg/kg) groups. Shown by Von Frey tests, the 14‐day oral application of DHA attenuated the allodynia behaviors in dose‐dependent manner (*R* ^2^ = 0.8508) (Figure 1C). Within this range, the‐L was almost ineffective, the‐M was equivalent to PGB, and the‐H achieved complete relief. The inhibitions of the central sensitization, shown by the rightwards shifts of curves showing the responses of the C‐fiber to the electrical stimulations, were achieved by DHA‐H and PGB, as compared with the SNL group (Figure 1C). The antidepressant effects, shown by the decreases of the immobility time in the forced swimming and tail suspension tests, were achieved by DHA‐M/H and DLT (Figure 1D). The anxiolytic effects, quantified by the open field test, were achieved by DHA‐M/H, but not DLT nor PGB (Sup. 1A).

TNFα has been reckoned as one of the prominent drug targets when dealing with NP.^4^ After 14‐day oral given, DHA‐H was proved to downregulate the expression of TNFα in both the SP and CA3 of SNL mice (Figure 1E); downregulate TNFα (*R* ^2^ = 0.7565) (Sup. 1B), attenuate the allodynia (*R* ^2^ = 0.6052), depressant (*R* ^2^ = 0.5108, 0.3903) and anxious (*R* ^2^ = 0.6914) (Sup. 1C) behaviors in mice injected with TNFα in the CA3 in the dose‐dependent manner (Figure 1F). Similarly, in mice injected with TNFα in the SP, 10‐day oral given of DHA was proved to downregulate TNFα (*R* ^2^ = 0.4825) (Sup. 1E) and C‐fiber transmitter cGRP (*R* ^2^ = 0.6343), attenuate the allodynia behaviours (*R* ^2^ = 0.4847) in dose‐dependent manners (Figure 1G). TNFα injection in the SP did not induce depression (Sup. 1D).

**FIGURE 2:**
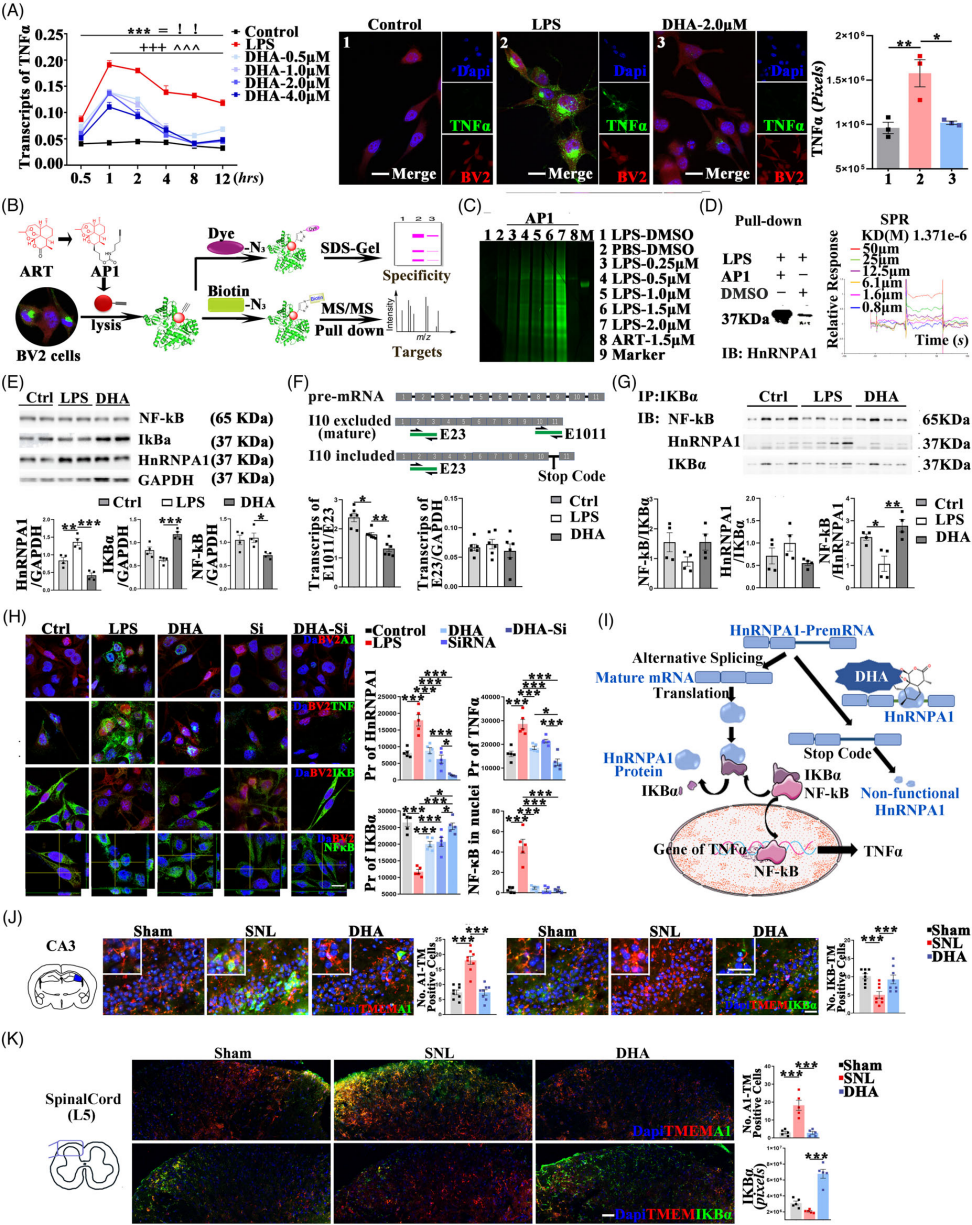
The binding and roles of HnRNPA1‐DHA in the regulation of the IKBα–NF‐kB–TNFα pathway. (A) Quantification of DHA mediated regulation of TNFα mRNA and protein (*n* = 3 wells of cell per group, scale bar 20 μm) in BV2 cells. The HnRNPA1‐DHA binding analysis include (B) the workflow, (C) the labelling profile, and (D) the pull‐down and SPR analyses. The HnRNPA1‐DHA mediated regulation analysis in BV2 cells include the quantification of the expression of HnRNPA1, IKBα, NF‐kB protein by (E) western blot (*n* = 4 wells of cell per group) and (H) fluorescence intensities (*n* = 5 wells of cell per group, scale bar 20 μm), (F) the expression of the transcripts with/without the exon10/11 splicing by RT‐PCR (*n* = 3 wells of cell per group), (G) the HnRNPA1–IKBα–NF‐kB competitive binding by co‐immunoprecipitation analysis (*n* = 4 wells of cell per group), and (I) summarized in the pattern diagram. The HnRNPA1‐DHA mediated regulation analysis in spinal cord ligation (SNL) mice were quantified in both (J) CA3 (*n* = 8 mice per group) and (K) SP (*n* = 5 mice per group) (scale bar 50μm). All data are shown by mean ± SEM *P < 0.05, **P < 0.01, ***P < 0.001

Probe‐based chemical proteomics experiments were designed to identify molecules underling DHA‐mediated downregulation of TNFα. In BV2 cells, 2μM DHA was proved sufficient to downregulate the lipopolysaccharide (LPS) induced overexpression of TNFα on both mRNA and protein levels (Figure 2A). The AP1 probe, which harbors the core structure and activity of ART,^5^ was used to label and identify the drug binding proteins (Figure 2B). AP1‐fluorescent dye was used to clarify the activity and specificity of probe (Figure 2C); AP1‐Biotin was used to label and purify the probe‐binding proteins as previously described.^6^ Among the 333 binding proteins, HnRNPA1 were identified as the top hub by the protein–protein interaction analysis (Sup. 2). The binding between DHA and HnRNPA1 was confirmed by the pull‐down and the surface plasmon resonance test (SPR) (Sup. 3). One of the widely acknowledged functions of HnRNPA1 is the degradation of IKBα, which contribute to the maximal activation of the NF‐kB dependent transcription of TNFα.^7^ In BV2 cells and SNL mice (SP and CA3), the DHA‐mediated downregulation of HnRNPA1 was proved and companied by the upregulation of IKBα (Figure 2E, H, J, K). The expression of HnRNPA1 is regulated in a precise way. In Bv2 cells, LPS‐induced overexpression of HnRNPA1 was proved to be accompanied by the decreases of the mature HnRNPA1 mRNA (E1011 included, Figure 2F). Further decreases of the mRNA containing E1011 were achieved by DHA, as compared with LPS. Further, clarified by the Co‐immunoprecipitation assay, the enhancement of the IKBα–NF‐kB binding and the weakness of the IKBα–HnRNPA1 binding was found by DHA, as compared with LPS (Figure 2G). The DHA–HnRNPA1 binding may (1) downregulate HnRNPA1 by a negative feedback regulation of E1011 splicing; (2) inhibit the transcription of TNFα by the regulations of both HnRNPA1 transcription and the HnRNPA1–IKBα–NF‐kB bindings (Figure 2I).

**FIGURE 3:**
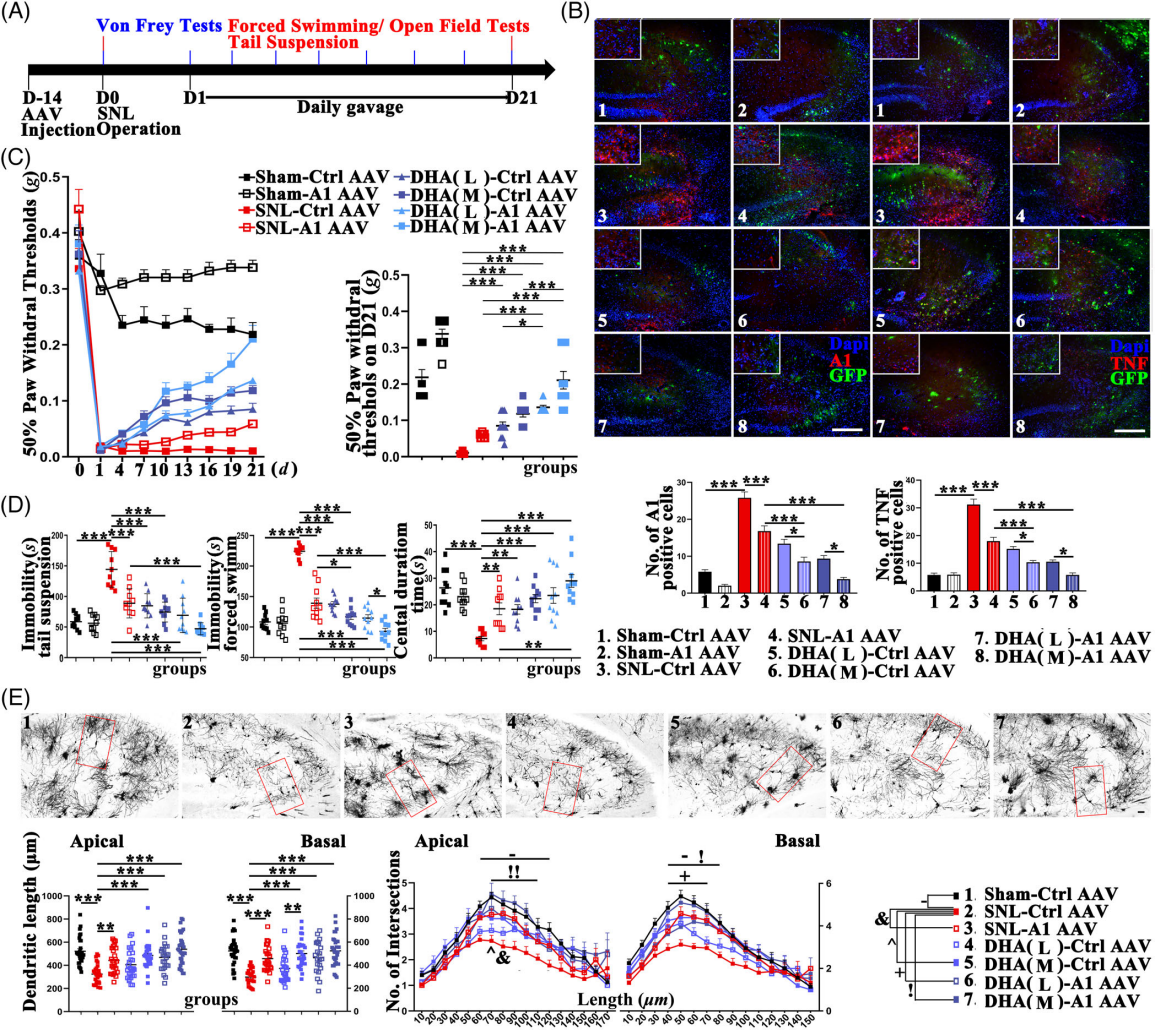
The remission of the neuropathic pain (NP) syndromes by the downregulation of HnRNPA1 in the CA3 of the spinal cord ligation (SNL) mice. (A) The time axis of the injections and the following tests. The curative effects are quantified by (B) the number of the HnRNPA1/TNFα positive cells in hippocampus (*n* = 6 mice per group, scale bar 100 μm), (C) the 50% withdrawal threshold in the Von Frey test, (D) immobility and central duration time in the tail suspension/forced swimming tests and the open field test (*n* = 10 mice per group), (E) the dendritic length and intersections of the CA3 pyramidal neurons (*n* = neurons from 4 mice per group, scale bar 10 μm). All data are shown by mean ± SEM *P < 0.05, **P < 0.01, ***P < 0.001

**FIGURE 4:**
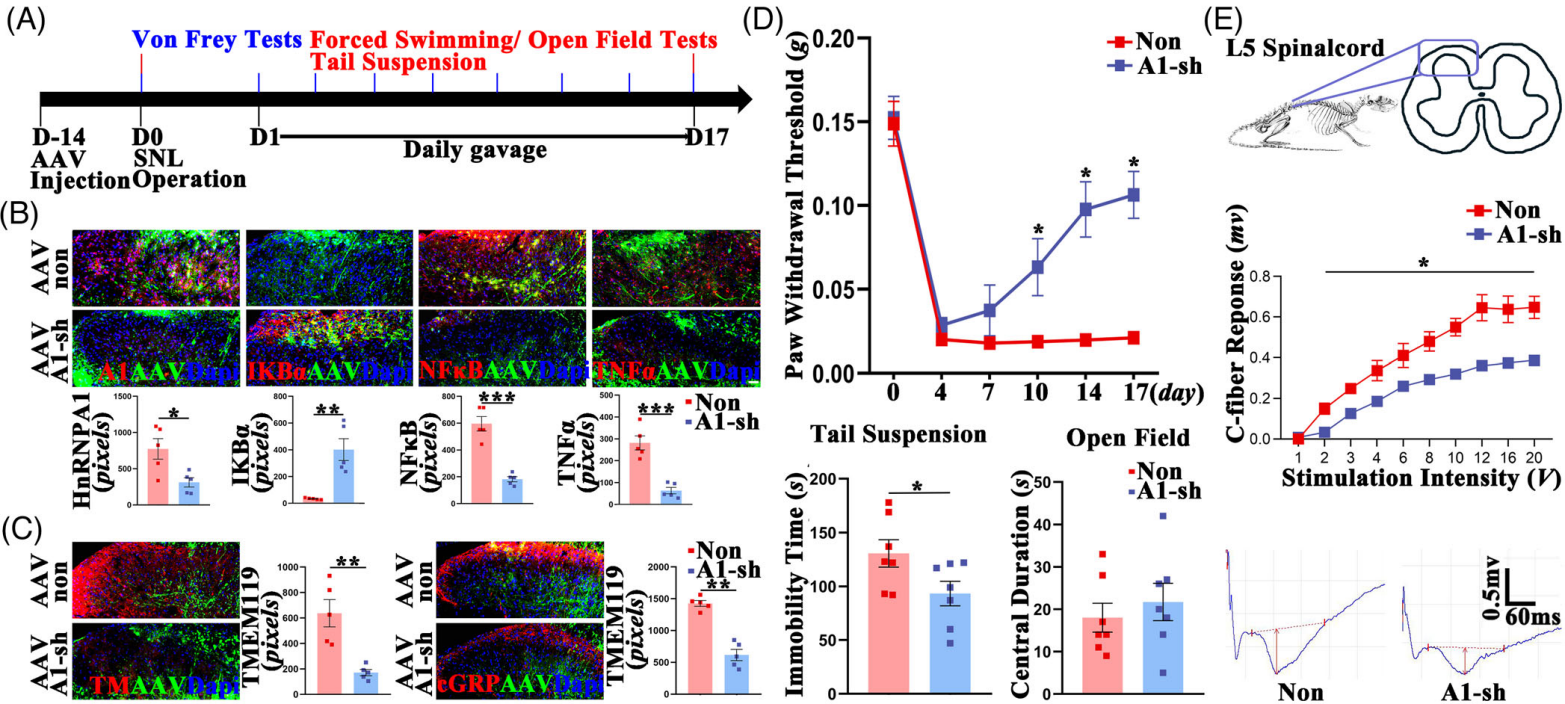
The remission of the neuropathic pain (NP) syndromes by the downregulation of HnRNPA1 in the SP of the spinal cord ligation (SNL) mice. (A) The time axis of the injections and the following tests. The curative effects are quantified by (B) the immunostaining of HnRNPA1, IKBα, NF‐kB and TNFα (*n* = 5 mice per group, scale bar 50 μm), (C) the immunostaining of microglia marker TMEM119 and C‐fiber transmitter cGRP (*n* = 5 mice per group, scale bar 50 μm), (D) 50% withdrawal threshold in the Von Frey test, immobility and central duration time in the tail suspension/forced swimming tests and the open field test (*n* = 7 mice per group), (E) the C‐fibre long‐term potentiation (LTP) (*n* = 8–12 mice per group). All data are shown by mean ± SEM *P < 0.05, **P < 0.01, ***P < 0.001

The following experiments were designed to clarify the associations between the downregulations of HnRNPA1 in the CA3/SP and the curations of NP (Figures 3A and 4A). In CA3, HnRNPA1 was downregulated by DHA‐L/M, adeno‐associated virus (AAV) encoding HnRNPA1 shRNA (A1‐AAV), and further downregulated by DHA‐L/M‐A1‐AAV (Figure 3B). In accordance with this, DHA and A1‐AAV had synergic effects on the down regulation of TNFα. The expressions of HnRNPA1 and TNFα were strongly associated (*R* ^2^ = 0.7781, Figure 3B). Shown by the results of the morphological and behavioral tests, the repairs of the CA3 pyramidal neurons and the remissions of the pain‐emotion syndromes were achieved by the DHA‐L/M and A1‐AAV. The joint usage of DHA‐M/H‐A1‐AAV showed better co‐curations as compared with the solo DHA and A1‐AAV (Figure 3C–E).

Similar, AAV encoding HnRNPA1 shRNA (A1‐sh) resulted in the downregulation of both HnRNPA1 and TNFα as compared with ones injected with the nonsense shRNA (Non) (Figure 4B). Further, A1‐sh resulted in significant downregulations of cGRP (Figure 4C), increases of pain threshold (Figure 4D), and decreases of C‐fibre LTP (Figure 4E) as compared with the Non. Shown by the decreases of the immobility time in the tail suspension and forced swimming test as compared with Non, A1‐sh were proved to be antidepressant (Figure 4D).

In conclusion, findings in this work may contribute to a better understanding of the mechanism underlying DHA mediated co‐curation of NP, as well as the roles HnRNPA1 assumed in the pain‐emotion comorbidity. Findings point out that the synergic regulations of the neuroinflammation in SP and CA3 may deal with NP on not only the sensory but also the emotional aspects.
